# Polycarbonate-Based Copolymer Micelles as Biodegradable Carriers of Anticancer Podophyllotoxin or Juniper Extracts

**DOI:** 10.3390/jfb15030053

**Published:** 2024-02-21

**Authors:** Radostina G. Kalinova, Ivaylo V. Dimitrov, Diana I. Ivanova, Yana E. Ilieva, Alexander N. Tashev, Maya M. Zaharieva, George Angelov, Hristo M. Najdenski

**Affiliations:** 1 Institute of Polymers, Bulgarian Academy of Sciences, 1113 Sofia, Bulgaria; kalinova@polymer.bas.bg (R.G.K.); dimitrov@polymer.bas.bg (I.V.D.); 2 Institute of Chemical Engineering, Bulgarian Academy of Sciences, 1113 Sofia, Bulgaria; 3 The Stephan Angeloff Institute of Microbiology, Bulgarian Academy of Sciences, 1113 Sofia, Bulgaria; illievayana@gmail.com (Y.E.I.); zaharieva26@yahoo.com (M.M.Z.); hnajdenski@abv.bg (H.M.N.); 4 Department of Dendrology, University of Forestry, 1797 Sofia, Bulgaria; altashev@mail.ru

**Keywords:** *Juniperus* L., podophyllotoxin, anticancer activity, nanocarriers, PEG–polycarbonate block copolymer micelles

## Abstract

Podophyllotoxin (PPT) is used in the industrial production of efficient anticancer, antiviral and other drugs. *Sinopodophyllum hexandrum* or *Podophyllum peltatum* are natural sources of PPT, but at present they are considered as endangered species. Their PPT content is variable, depending on the growing conditions. Searching for new sources of PPT, some representatives of the genus *Juniperus* were found to exhibit efficient PPT biosynthesis. However, PPT is highly toxic and poorly soluble in water compound, which limits its clinical applications. In this connection, amphiphilic polymer micelles are considered to be suitable PPT carriers, aimed at increase in water solubility and decrease in toxicity. The present research deals with the evaluation of MPEG–polycarbonate block copolymer micelles loaded with PPT or juniper extracts. The active component-loaded polymer nanocarriers were characterized by dynamic and electrophoretic light scattering, as well as by transmission electron microscopy. The active component loading efficiency and loading capacity were also determined. Highly efficient antiproliferative activity of the loaded micelles was determined in a panel of cancer cell lines. The obtained amphiphilic nanocarriers, loaded with PPT-containing bioactive components, have application in future in vivo preclinical trials of their pharmacokinetics and pharmacodynamics as potential therapeutical agents in the prospective nanomedicine.

## 1. Introduction

Nanotechnology reveals new approaches towards therapy for cancer and other diseases [1]. Nanoparticles have dimensions of the order of nanometers to microns and are small compared to the cells; however, bioactive molecules can be incorporated in their structures and transported into the cells [2]. Nanomaterials have been considered to improve the solubility and reduce the adverse effects of many phytochemicals or synthetic drugs, approved for cancer treatment [3,4,5,6]. Efficient anticancer drugs have been loaded into nanosized carriers and used for treatment of various forms of cancer. For example, albumin-bound paclitaxel (nab-paclitaxel, Abraxane^®^) was approved for therapy for breast, lung and pancreatic cancer. Liposome incorporated doxorubicin (Doxil^®^, Myocet^®^, Caelyx^®^, etc.) is an antitumor antibiotic, which demonstrated less cardiotoxicity and was approved for therapy for various forms of cancer [7].

**Figure 1:**
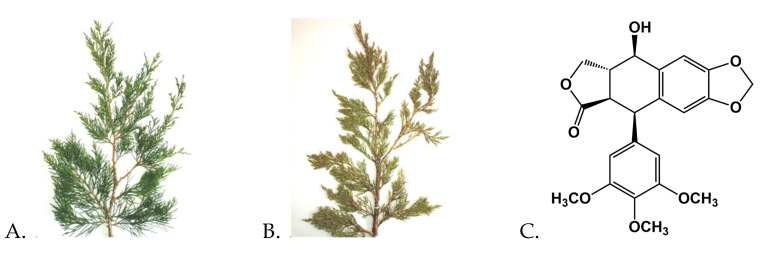
Specimens of *Juniperus virginiana* (**A**) and *Juniperus sabina* var. *balkanensis* (**B**), whose leaf extracts, containing podophyllotoxin (**C**), were used for loading into biodegradable amphiphilic MPEG-*b*-PC diblock copolymer nanocarriers.

Podophyllotoxin (PPT) is used in the industry as a precursor for production of anticancer, antiviral, anthelminthic and other drugs [8]. PPT is obtained from *Podophyllum peltatum* L. (American Mayapple) or *Sinopodophyllum hexandrum* (Royle) T. S. Ying (Indian Mayapple) [9], which are already endangered species because of their intensive use in the industry. In addition, their PPT biosynthesis is dependent on their cultivation conditions [10]. Therefore, other plant species have been screened for detection of PPT and its derivatives. Junipers are evergreen plants that exhibit stable seasonal biosynthesis of PPT [11,12]. Using UHPLC/HRMS analysis, representatives of the genus *Juniperus* L., including the species studied here (Figure 1), were determined previously to contain efficient concentrations of PPT and other anticancer lignans, and demonstrated high antiproliferative activity in various cancer cell lines. For example, the *Juniperus virginiana* L. leaf extract was found to contain PPT (0.4%), as well as deoxypodophyllotoxin, β-peltatin, anhydropodorhizol and matairesinol; *Juniperus sabina* var. *balkanensis* R.P. Adams and A.N. Tashev leaf extract was determined to contain PPT (0.2%), as well as deoxypodophyllotoxin, anhydropodorhizol and yatein [13]. The efficient activity of the studied extracts was supposed to be due to the combined activity of the detected anticancer compounds.

PPT acts effectively against some human papilloma viruses (HPV), which are DNA viruses belonging to the *Papillomaviridae* family, causing various warts, papillomas, oropharyngeal or genital cancers, etc. For example, Podofilox^®^ (0.5% PPT content) is a drug against genital warts (*Condyloma acuminatum*). PPT acts as a DNA-replication inhibitor, but is a poorly water-soluble compound with high toxicity. Structural modifications of PPT, aimed at increasing its water solubility, led to the discovery of the drugs Etoposide^®^, Etoposide phosphate^®^ and Teniposide^®^, used at present as gold standards for therapy for various forms of cancer. Codelivery of PPT or Etoposide with other efficient anticancer drugs (paclitaxel, doxorubicin, cisplatin, all-*trans-*retinoic acid, vorinostat, etc.) loaded into various nanocarriers, such as polymeric micelles, vesicles etc., was aimed at improved drug pharmacokinetics and synergy [14].

Amphiphilic block copolymers with hydrophilic and hydrophobic segments can form micelles, which can increase the solubility of poorly water-soluble substances by inclusion of the corresponding molecules in their hydrophobic nucleus. As a consequence, the obtained nanomaterials can reduce the toxicity of the loaded substances by facilitating their transport to the cells, permeability across biomembranes and consequent excretion from the living organisms [15]. In this connection, poly(ethylene glycol) (PEG) is the most commonly used hydrophilic component [16], while polylactide (PLA), poly(ε-caprolactone) (PCL), hydrophobic poly(amino acids), aliphatic polycarbonates, etc., are suitable hydrophobic components [17,18].

PPT was successfully loaded into micelles formed from a graft copolymer PASP-*g*-ODA-*g*-PEG, obtained by linking a hydrophilic monomethoxy poly(ethylene glycol) segment to a hydrophobic poly(aspartic acid-*g*-octadecyl-amine) main chain [19]. In another experiment, PPT was loaded by entrapment through hydrophobic interaction in the core of micelles, formed from modified copolymer PLG-*g*-mPEG (poly(*L*-glutamic acid)-*graft*-methoxy poly(ethylene glycol)) with high loading efficiency (94%) and drug loading content (28.2 wt%). The obtained PPT-loaded micelles exhibited efficient antiproliferative activity in A549 (non-small-cell lung cancer) cells [20].

Deoxypodophyllotoxin (DPT) was loaded with 99% efficiency into polymeric micelles of methoxy poly(ethylene glycol)-*block-*poly(D,L-lactide) (MPEG-PLA). The DPT-loaded polymeric micelles compared to the starting materials showed higher cytotoxicity in HeLa cells, as well as prolonged plasma circulation time and improved plasma clearance rate in in vivo studies. The obtained micelles showed a pH dependent selective drug release in the acidic environment of the tumor cell cytosol (whereas pH of healthy cells was considered to be in the neutral region) [21].

In other studies, polymer–drug conjugates were obtained as potential anticancer therapeutics [22]. For example, epipodophyllotoxin was covalently bound between hydrophilic and hydrophobic chains, forming micelle-drug conjugates with efficient antitumor properties [23]. Alternatively, a nanomaterial, consisting of polymeric micelles containing PPT linked by an ester linkage to polyacrylic acid (PAA), showed increased cytotoxicity in breast cancer cells (MCF-7, MDA-MB-231) and decreased cytotoxicity in normal HEK-293 cells in comparison with the starting materials [24].

**Figure 2:**
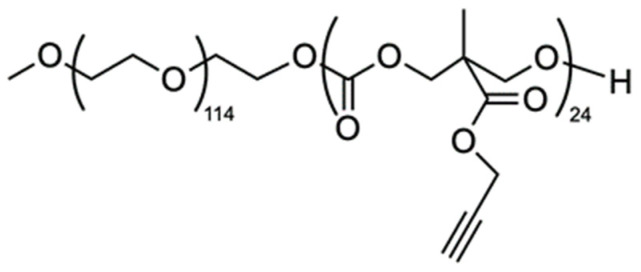
Structure of a biodegradable amphiphilic MPEG-*b*-PC diblock copolymer, used in this study to obtain podophyllotoxin- or juniper extract-loaded nanocarriers.

A biocompatible and biodegradable amphiphilic block copolymer MPEG-*b*-PC [methoxy poly(ethylene glycol)-*block-*polycarbonate] (Figure 2) was obtained recently by green polymer synthesis and its curcumin-loaded micelles were formed by co-association in aqueous media [25].

The present study was aimed at determination of the physico-chemical characteristics, bioactive component loading properties and antiproliferative activity of poly(ethylene glycol)-*block-*polycarbonate (MPEG-*b*-PC) based micelles loaded with PPT or cytotoxic *Juniperus virginiana* (JV) or *Juniperus sabina* var. *balkanensis* (JS) leaf extracts. The obtained nanomaterials, containing hydrophilic corona and hydrophobic core, loaded with PPT or antiproliferative juniper extracts, were considered as potential pharmaceutical agents for therapy for various diseases, including benign or malignant tumors, HPV-infections etc.

## 2. Materials and Methods

### 2.1. Chemicals

Podophyllotoxin (standard compound, ≥98%), MTT [3-(4,5-dimethylthiazol-2-yl)-2,5-diphenyltetrazolium bromide], *L-*glutamine, ethanol (96%), acetone (ACS reagent, ≥99.5%) and acetonitrile (99%) were purchased from Sigma-Aldrich Fine Chemicals (St. Louis, MO, USA). Methanol, 2-propanol and formic acid were from Chimspectar Ltd., Sofia, Bulgaria. Cell culture media DMEM, RPMI-1640, IMDM, fetal bovine serum (FBS) and Penicillin/Streptomycin (Pen/Strep, 100×) were delivered from Capricorn Scientific (Düsseldorf, Germany).

### 2.2. Plant Materials and Extracts Preparation

The plant species were authenticated by A. N. Tashev and voucher specimens were deposited in the Herbarium of the Institute of Biodiversity and Ecosystem Research, Bulgarian Academy of Sciences (SOM). *Juniperus virginiana* (SOM 174406) was obtained (11 May 2023) from the Arboretum of the University of Forestry, Sofia. *Juniperus sabina* var. *balkanensis* (SOM 177009) was collected (11 May 2023) from a protected area Gyumyurdzhynski Snezhnik in the region of peak Veykata (1463 m a.s.l.), Eastern Rhodopes, Bulgaria.

Plant extracts were obtained by a modified method [13]. Briefly, dry ground juniper leaves were suspended (liquid/solid ratio 10 *v*/*w*) in methanol (80% *v*/*v*) in an Erlenmeyer flask with a stopper. The extraction was carried out in a shaker water-bath for 4.5 h at room temperature. The extract was collected by filtration and methanol was evaporated in vacuo. During evaporation, a chlorophyll containing dark green oil appeared on the flask walls and was removed. The remaining water containing extract was freeze-dried (24 h, −50 °C, 0.1 mbar) and the crystallized product was stored in the freezer (−20 °C).

### 2.3. Polymer Synthesis

The amphiphilic block copolymer MPEG-*b*-PC was synthesized according to a previously reported procedure [25]. Briefly, the ring-opening polymerization of an alkyne-functional cyclic monomer was initiated by a methoxy-poly(ethylene glycol) (MPEG) macroinitiator using 4-(dimethylamino)pyridine as a catalyst and was performed in bulk at 60 °C. ^1^H-NMR (600 MHz, DMSO-d6, δ, ppm): 4.73 (s, OC*H*_2_C≡CH), 4.21–4.26 (m, OCH_2_C*H*_2_O(C=O) + OC(O)OC*H*_2_), 3.50 (s, OC*H*_2_C*H*_2_O), 3.23 (s, C*H*_3_O), 2.52 (s, CH_2_C≡C*H*) and 1.18 (s, C*H*_3_).

### 2.4. Preparation of Micelles

The nanosized micelles were formed through a self-assembly of the block copolymer according to the following procedure: first, the copolymer was dissolved in acetone and its concentration was adjusted to 10 mg mL^−1^. Afterwards, the copolymer solution (1 mL) was added slowly via a syringe to vigorously stirred (1000 rpm) ultrapure water (~8 mL). The acetone was evaporated in vacuo and ultrapure water was added to the micellar dispersion to adjust the concentration to 1 mg mL^−1^. Prior to use, the micellar dispersions were filtered (Millipore^®^ 0.45 μm) and subjected to characterization.

### 2.5. Drug Loading of Micelles

For the preparation of loaded micelles, the bioactive components podophyllotoxin (PPT) or juniper extracts (JV and JS) were firstly dissolved in ethanol at a concentration of 1 mg mL^−1^ and then 1 mL of this solution was added slowly to 10 mL (1 mg mL^−1^) of the stirred MPEG-*b*-PC micelles’ dispersion. After removal of ethanol in vacuo, the concentration was adjusted to 1 mg mL^−1^ (micelles to PPT or juniper extracts ratio—10:1 *w*/*w*). The micellar dispersions were filtered (0.45 µm), lyophilized and then resuspended in acetonitrile (PPT-loaded micelles) or ethanol (juniper extract-loaded micelles) for UV/Vis spectroscopic analysis at wavelength of 274 nm and 277 nm, respectively.

The extinction coefficient ε = 4200 M^−1^ cm^−1^ (λ_max_ = 274 nm) of PPT in acetonitrile was obtained from the calibration curve. The extinction coefficients ε = 5.5009 L g^−1^ (λ_max_ = 277 nm) of juniper extract JS and ε = 5.2355 L g^−1^ (λ_max_ = 278 nm) of juniper extract JV in ethanol were obtained from the corresponding calibration curves. These values were used to calculate the quantity of the active substance encapsulated into the micelles. The loading efficiency (LE) and loading capacity (LC) were calculated according to the following equations [26]:

LE (wt%) = AS_1_/AS_0_ × 100 (1)

LC (wt%) = AS_1_/M × 100 (2)

where AS_1_ is the amount of the active substance in micelles, AS_0_ is the total initial amount of the active substance, and M is the amount of the micelles (active substance plus polymer).

### 2.6. Methods for Characterization of Micelles

The micelles’ morphology and sizes were observed by Transmission electron microscope HRTEM JEOL JEM-2100 (200 kV) (Peabody, MA, USA) with CCD camera GATAN Orius 832 SC1000 (Pleasanton, CA, USA) using GATAN Microscopy Suite 3.4 Software (Gatan, Inc., Pleasanton, CA, USA). UV/Vis spectra were recorded on a DU 800 Beckman Coulter spectrometer (Brea, CA, USA). Dynamic light scattering by a NanoBrook Plus PALS instrument (Brookhaven Instruments, New York, NY, USA) was used for measurement of the particles’ mean diameters, polydispersity indexes (PdIs), and zeta potentials in fully hydrated state. The instrument was equipped with a 35-mW solid-state laser operating at λ = 660 nm and at a scattering angle of 90°.

The Stokes–Einstein equation was applied for detection of particles’ hydrodynamic diameters (*d_H_*):

*d_H_ = kT/(3πηD)* (3)

where *k* is the Boltzmann’s constant, *T*—absolute temperature, *η*—viscosity, and *D*—diffusion coefficient.

The Smoluchowski equation was used for calculation of *ζ*-potentials from the detected electrophoretic mobility:

*ζ = 4πημ/ε* (4)

where *η* is the solvent viscosity, *μ*—electrophoretic mobility, and *ε*—dielectric constant of the solvent.

The detections of the size and zeta potentials were performed in triplicate and were presented as averages of 3 and 20 runs, respectively.

### 2.7. Cell Culture Conditions

The antiproliferative activity of empty and drug-loaded nanocarriers was studied using a panel of cell lines: MJ (cutaneous T cell lymphoma), A549 (human lung carcinoma), MDA-MB-231 [triple (ER, PR, HER2) negative breast adenocarcinoma], and the non-tumorigenic cell line HaCaT (immortalized human keratinocytes). MDA-MB-231 was from the German Collection of Microorganisms and Cell Cultures (DSMZ GmbH, Braunschweig, Germany). The rest of the cell lines were from American Type Culture Collection (ATCC, Manassas, VA, USA). The cells were maintained in humidified incubator (Panasonic MCO-18AC, Panasonic Healthcare Ltd., Oizumi-Machi, Japan) at 37 °C and 5% CO_2_. The growth medium for A549 and HaCaT cells was DMEM-HG with 4.5 g/L glucose, for MDA-MB-231*–*RPMI-1640, and for MJ*–*IMDM. The cell culture medium was supplemented with a heat-inactivated fetal bovine serum (FBS): 20% FBS for IMDM, and 10% FBS for the other media. *L*-glutamine (4 mM) and Pen/Strep (Penicillin G Sodium 10^5^ Units/L and Streptomycin Sulfate 100 mg/L, final concentrations) were also added to all cell culture media. MJ cells were grown in a suspension, while all the other cell lines were adherent. The MJ cells were passaged 2–3 times per week and the adherent cell lines were trypsinized 1–2 times per week, which allowed the cells to remain in the log phase.

### 2.8. MTT-Assay for Cytotoxicity

MTT-assay was carried out according to a modified protocol of Annex C, ISO 10993-5 [27,28]. In summary, 96-well plates with a flat bottom were seeded with 100 µL of the cell suspension at a concentration of 3 × 10^5^ cells/mL for MJ, and 10^5^ cells/mL for the other cell lines. The cells were incubated for 24 h to reach the log phase of growth. Then the cells were treated with PPT or juniper extracts, or with the corresponding drug-loaded or empty micelles, and were incubated for an additional 72 h. After that, MTT solution (10 µL, 5 mg/mL) was added to each well and then the plates were maintained at 37 °C for two hours. Subsequently, for the adherent cell lines, the medium was aspirated and the formed formazan crystals were dissolved in 2-propanol (100 μL per well). For the suspension cells, 5% formic acid was added to 2-propanol (100 μL per well). As a blank sample, either 100 μL of 2-propanol (for the adherent cells) or a mix of cell culture medium (100 μL), MTT solution (10 μL) and 2-propanol (100 μL) with 5% formic acid (for the suspension cells) was used. Untreated cells were the negative control. The reference filter was set at 690 nm and the absorption was measured at 540 nm using a microplate reader (MicroTek Instruments, Inc., Winooski, VT, USA). The cell viability of the treated cells was calculated as a percentage of the untreated control cells. MTT-assays were carried out using at least four wells, seeded for each concentration of the corresponding sample.

### 2.9. Data Processing and Statistics

A non-linear regression analysis (Curve fit, GraphPad Prizm 6.01 software, GraphPad Software Inc., San Diego, CA, USA) was used to calculate the IC_50_ values from the MTT assays using sigmoidal concentration–response curves. For statistical analysis, Student’s *t*-test was used with *p* ≤ 0.05 set as the lowest level of statistical significance.

## 3. Results and Discussion

### 3.1. Preparation and Drug Loading of Micelles

The poly(ethylene glycol)-*b*-polycarbonate (MPEG-*b*-PC) diblock copolymer was successfully synthesized through bulk ring-opening polymerization of an alkyne-functional cyclic carbonate monomer initiated from the methoxy-poly(ethylene glycol) macroinitiator. The polymerization process was carried out in an inert atmosphere at 60 °C.

The obtained biodegradable and biocompatible polymer was further used for the preparation of nanoparticles intended for bioactive substance delivery. First, amphiphilic MPEG-*b*-PC macromolecules were self-assembled into spherical micelles via nanoprecipitation method using an acetone–water system. The obtained micelles comprising hydrophobic core and a hydrophilic external layer were loaded with three efficient cytotoxic agents—podophyllotoxin or two different podophyllotoxin-containing juniper extracts according to a method involving absorption of the drug after the micelles’ formation [29,30,31]. The active substances were added as ethanolic solutions to the aqueous dispersions of the preformed micelles followed by evaporation of ethanol. The concentration of the drug loaded micellar dispersions was adjusted to 1 mg mL^−1^ and the unloaded free water-insoluble drugs were removed through filtration (Millipore filter, 0.45 μm pore size).

In order to determine encapsulation efficiency of PPT and juniper extracts in MPEG-*b*-PC micelles, predetermined amounts of micelles loaded with active substances were lyophilized and the solid residues were resuspended in acetonitrile (for PPT-loaded micelles) or ethanol (for extract-loaded micelles). The UV/Vis spectroscopy was then applied to quantify the active substance content in the micelles. The LE and LC were calculated according to Equations (1) and (2) and the results were summarized in Table 1. The JV-loaded micelles were characterized with LE of 62 wt% and a LC of 9.9%. A significantly higher encapsulation efficiency values at a quantitative level were obtained for PPT- and JS-loaded polymer micelles. This loading efficiency corresponded to a previously found 99% loading efficiency of deoxypodophyllotoxin into MPEG-PLA methoxy poly(ethylene glycol)-block-poly(*D*,*L*-lactide) micelles. The corresponding LC values for PPT- and JS-loaded polymer micelles in the present study were 10.8 and 14.7%, respectively. Higher loading efficiency in the obtained amphiphilic micelles was considered to be due to the higher hydrophobic properties of the loaded sample. Thus, the detected lower loading efficiency of JV leaf extract in comparison with the JS leaf extract is most likely to be due to the difference in the complex chemical composition, especially in its hydrophobic part, of the studied extracts.

### 3.2. Physico-Chemical Characterization of Micelles

**Figure 3:**
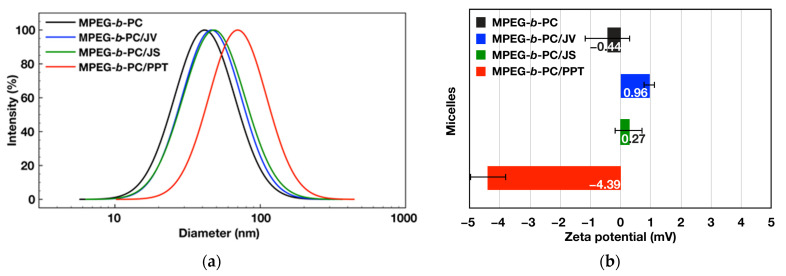
Presentation of the size distributions (**a**) and zeta potentials (**b**) obtained from dynamic and electrophoretic light scattering analyses of the corresponding micelles: empty (MPEG-*b*-PC: d = 44 nm, PdI 0.245, ζ = −0.44 mV), JV-extract loaded (MPEG-*b*-PC/JV: d = 46 nm, PdI 0.246, ζ = 0.96 mV), JS-extract loaded (MPEG-*b*-PC/JS: d = 48 nm, PdI 0.272, ζ = 0.27 mV) and PPT-loaded (MPEG-*b*-PC/PPT: d = 70 nm, PdI 0.226, ζ = −4.39 mV) block copolymer micelles.

As the particle size is one of the most crucial factors influencing material transport across the cell membrane, it is fundamental to determine the hydrodynamic diameter of nanomaterials in fully hydrated state. The aqueous dispersions of both empty and loaded with active components micelles were analyzed by DLS. The average particle diameter of the empty MPEG-*b*-PC micelles was found to be 44 nm with relatively narrow size distribution (polydispersity indices PdI = 0.257) (Table 1 and Figure 3a).

The PPT-loaded micelles were around 70 nm in diameter with PdI less than 0.3. The results showed an approximately 26 nm increase in the average diameters for the PPT-loaded micelles in comparison with the empty micelles. The notable size increase could be attributed to the micelles’ core expansion as a result of the drug accommodation. On the contrary, loading the polymer micelles with juniper extracts leads to insignificant increase in particle size as detected by DLS measurements. The average diameters increased to 46 and 48 nm for JV and JS loaded micelles, respectively. This is an indication that the incorporation of both extracts did not disrupt the structure of micelles. Moreover, the previously reported results revealed that drug formulations with average diameters of approx. 50 nm show the highest tumor tissue retention integrated over time and more efficient cancer cell internalization [32], thus making MPEG-*b*-PC micelles loaded with juniper extracts attractive for potential biomedical applications.

Finally, the measured zeta potentials close to 0 mV for both unloaded and loaded micelles confirmed the effective surface shielding of nanoparticles by a neutral, hydrophilic PEG corona (Figure 3b). Hydrophobic interactions were endorsed as responsible for loading of the hydrophobic active substances, mainly in the hydrophobic core of the corresponding micelles. However, small changes in the particles’ zeta potentials after the active compounds loading were observed. A small negative zeta potential of the PPT-loaded micelles was attributed to the specific interactions between the polymer micelles and PPT molecules, containing hydroxyl and other functional groups. Small changes in the particles’ zeta potentials were most likely to be due to the presence of low amounts of active components located closely to the border between the core and the shell of the micelles formed from PEG-*b*-PC copolymer, and hence are more mobile and partially adsorbed on the micelles’ surface.

**Figure 4:**
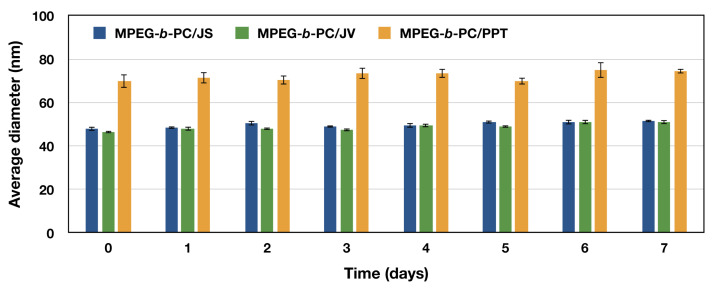
Stability of the copolymer micelles loaded with JS-leaf extract, JV- leaf extract or PPT, assessed by DLS measurements in aqueous media after various time intervals of incubation at 4 °C.

In addition, the stability of the loaded polymeric micelles was examined over a period of time and the changes in particle size and size distribution were followed by DLS. The results indicated that the particles were stable for at least one week in the refrigerator (Figure 4).

**Figure 5:**
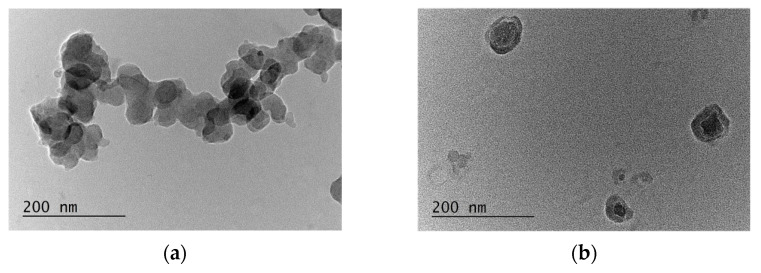

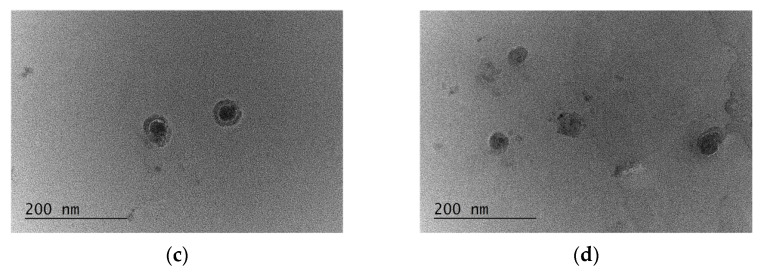
Transmission electron microscopy images of the corresponding micelles: (**a**) empty (d = 45.87 ± 4.68 nm); (**b**) PPT-loaded (d = 71.35 ± 5.14 nm); (**c**) JS-extract loaded (d = 53.18 ± 2.58 nm); and (**d**) JV-extract loaded (d = 46.96 ± 6.89 nm) block copolymer (MPEG-*b*-PC) micelles.

The morphology (size and shape) of empty and loaded block copolymer micelles was evaluated by transmission electron microscopy (TEM). The results presented in Figure 5 clearly indicated the spherical shape of nanoparticles, with average diameters close to those obtained from the DLS-measurements.

### 3.3. Antiproliferative Activity Analyses of Empty and Drug-Loaded Nanocarriers

The antiproliferative properties of bioactive component-loaded and empty nanocarriers were analyzed on a panel of cancer and normal cell lines (Table 2). The corresponding half-maximum growth-inhibitory concentrations (IC_50_) were calculated from the dose–response curves (Supplementary Material) derived from the MTT-assay of cancer and normal cell lines treated with polymer micelles loaded with podophyllotoxin or various juniper extracts. Lower IC_50_ value indicated higher activity of the sample.

For the MTT-assays, the following cancer cell lines were selected: A549 human lung carcinoma, MJ cutaneous T cell lymphoma, and MDA-MB-231 breast adenocarcinoma. MDA-MB-231 cells were used as a model for triple negative (ER, PR, HER2 negative) advanced breast cancer where the cellular control of the estrogen (ER) and progesterone (PR) receptors is lost and the cells are non-sensitive to hormone therapy. HaCaT cell line was used as a model for cytotoxicity analyses on normal human epidermal keratinocytes.

The results showed that the bioactive component-loaded nanocarriers exhibited efficient cytotoxicity in all studied cancer and normal cells, whereas the empty nanocarriers showed no cytotoxicity at the studied concentrations up to 40 μg/mL. Highest antiproliferative activity was detected for PPT and PPT-loaded micelles in all cancer or normal cell lines. Further, JS-extract as well as the corresponding loaded micelles exhibited better activity (lower IC_50_ values) than the corresponding JV-extract and JV-loaded micelles (higher IC_50_ values) in the studied cell lines. Higher activity of the JS-loaded micelles (in comparison with the JV-loaded nanocarriers) was attributed to higher activity of the corresponding extract, as well as to its higher loading efficiency (Table 1).

Generally, the average IC_50_ values of the loaded micelles showed higher (resp. lower IC_50_ values) or similar activities (IC_50_ near or in the same confidence interval) in comparison with the starting bioactive components. However, loading of the studied bioactive components and their solubilization in the core of amphiphilic micelles had the advantage of increased water-solubility of the obtained product in contrast with the initially water-insoluble PPT or juniper extracts. This effect was targeted at a change of the pharmacokinetic profile, reduced toxicity of the corresponding nanocarrier and facilitation of its excretion from the organisms in in vivo preclinical trials envisaged in future studies.

## 4. Conclusions

The obtained amphiphilic polymer micelles, containing hydrophobic polycarbonate core and hydrophilic PEG corona, were successfully loaded with PPT or antiproliferative juniper extracts. Hydrophobic interactions between the core and the active component contributed to the physico-chemical basis of the entrapment of the active component into the hydrophobic core of the obtained micelles. High encapsulation efficiency at a quantitative level was obtained for PPT- and *J. sabina* leaf extract (JS)—loaded polymer micelles. The corresponding LC values were 10.8 and 14.7%, respectively. The *J. virginiana* (JV) leaf extract—loaded micelles were characterized with LE of 62 wt% and a LC of 9.9%. In addition, the analysis of the size distribution of the nanoparticles showed that the PPT-loaded micelles were around 70 nm in diameter. On the other side, loading of the polymer micelles with juniper extracts led to insignificant increase in particle size (average diameters of 46 and 48 nm for JV and JS loaded micelles, resp.) in comparison with the empty nanocarriers (average diameter 44 nm). The polydispersity indices (PdI) values were less than 0.3. These results revealed that the MPEG-*b*-PC micelles loaded with juniper extracts are suitable candidates for potential biomedical applications, considering previously reported observations that drug agents with average diameters of approx. 50 nm show highest tumor tissue retention and efficient cancer cell internalization.

The analysis of the antiproliferative properties revealed that highest activity was detected for PPT-loaded micelles, followed by *J. sabina* var. *balkanensis* and *J. virginiana* leaf extract loaded nanocarriers in all cancer or normal cell lines. Higher activity of the JS-loaded micelles (in comparison with the JV-loaded nanocarriers) was attributed to higher activity of the corresponding extract as well as to its higher loading efficiency. The empty nanocarriers showed no cytotoxicity at the studied concentrations.

The comparison of the activity of the loaded nanocarriers with the starting bioactive components revealed that, generally, the average IC_50_ values of the loaded micelles showed higher (resp. lower IC_50_ values) or similar (IC_50_ near or in the same confidence interval) activities relatively to the individual components. However, loading of PPT or juniper extracts in the hydrophobic core of amphiphilic micelles solubilized the starting substances, which effect was targeted at a change in their pharmacokinetic properties, facilitating excretion and reduced toxicity of the obtained water-soluble product.

Further in vivo studies of the pharmacokinetics and pharmacodynamics, including excretion analyses and reduced toxicity of the loaded water-soluble nanocarriers, are envisaged in the future. In this connection, hemocompatibility and adverse effects analyses of the studied potential nanomedicines will be of great significance [33].

The obtained PPT- or juniper extract-loaded micelles with highly efficient antiproliferative activity are of substantial interest for forthcoming preclinical trials of the studied nanocarriers, with potential application in the prospective nanomedicine.

## Figures and Tables

**Table 1:** Characteristics of empty and active components of loaded block copolymer micelles.

Code	d ^a^ (nm)	PdI ^a^	ζ ^a^ (mV)	LE ^b^ (%)	LC ^b^ (%)
MPEG-*b*-PC	43.61 ± 0.42	0.257	−0.44 ± 0.75	-	-
MPEG-*b*-PC/PPT	69.98 ± 3.07	0.226	−4.39 ± 0.61	99.8 ± 0.9	10.8 ± 0.7
MPEG-*b*-PC/JS	47.75 ± 0.62	0.272	0.27 ± 0.46	99.6 ± 1.3	14.7 ± 1.0
MPEG-*b*-PC/JV	46.45 ± 0.44	0.246	0.96 ± 0.19	62.3 ± 0.7	9.9 ± 0.8

^a^ Abbreviations: average micelle diameters (d), size distributions (PdI) and zeta potentials (ζ) obtained from DLS measurements; PPT—podophyllotoxin; JS—*Juniperus sabina* var. *balkanensis* leaf extract; JV—*Juniperus virginiana* leaf extract; ^b^ Loading efficiency (LE) and loading capacity (LC) were spectroscopically determined.

**Table 2:** Antiproliferative properties of the bioactive component-loaded or empty nanocarriers in comparison with individual juniper leaf extracts or PPT in cancer or normal cell lines.

Sample	MDA-MB-231 IC_50_ (95% CI)	A549 IC_50_ (95% CI)	MJ IC_50_ (95% CI)	HaCaT IC_50_ (95% CI)
JV-extract	3.53 (2.92–4.27)	2.30 (1.97–2.68)	3.35 (2.49–4.51)	0.67 (0.64–0.70)
JV-loaded MC	2.78 (2.27–3.41)	2.25 (1.79–2.81)	1.24 (1.01–1.51)	0.57 (0.50–0.66)
JS-extract	0.55 (0.44–0.70)	0.42 (0.35–0.50)	0.25 (0.15–0.42)	0.09 (0.08–0.10)
JS-loaded MC	0.61 (0.51–0.73)	0.32 (0.24–0.41)	0.28 (0.20–0.38)	0.07 (0.06–0.08)
PPT	0.036 (0.022–0.059)	0.011 (0.008–0.015)	0.010 (0.007–0.014)	0.0012 (0.0011–0.0013)
PPT-loaded MC	0.023 (0.019–0.028)	0.017 (0.014–0.021)	0.014 (0.011–0.019)	0.004 (0.003–0.005)
Empty micelles *	>40	>40	>40	>40

Legend: MC-polymer micelles; JV—*J. virginiana* leaf extract; JS—*J. sabina* var. *balkanensis* leaf extract; PPT—podophyllotoxin; MDA-MB-231—triple negative (ER, PR, HER2 negative) breast adenocarcinoma cells; A549—human lung carcinoma cells; MJ—cutaneous T cell lymphoma cells; HaCaT—normal human epidermal keratinocytes; IC_50_ [μg/mL]—half-maximum growth-inhibitory concentration of the corresponding sample after treatment of the cancer cells, given in micrograms per milliliter of the cell culture medium. Lower IC_50_ values correspond to higher activities; 95% CI—confidence interval with regression coefficient (R^2^) in the range of 0.76–0.99; * no cytotoxicity up to 40 μg/mL.

## Data Availability

Data are contained within the article.

## Supplementary Materials

The following supporting information can be downloaded at: https://www.mdpi.com/article/10.3390/jfb15030053/s1, Dose–response curves derived from MTT-assays of cancer and normal cell lines treated with polymer micelles loaded with podophyllotoxin or various juniper extracts.
