# Supplementary material for: Polycarbonate-Based Copolymer Micelles as Biodegradable Carriers of Anticancer Podophyllotoxin or Juniper Extracts
Source: J Funct Biomater. 2024 Feb 21;15(3):53. doi: 10.3390/jfb15030053 (PMC10971044; doi:10.3390/jfb15030053)
Supplement: Supplementary file 1 [file jfb-15-00053-s001.zip › jfb-2822837-supplementary.pdf]

Supplementary

Polycarbonate-Based Copolymer Micelles as Biodegradable Carriers of Anticancer Podophyllotoxin or Juniper Extracts

Radostina G. Kalinova [1], Ivaylo V. Dimitrov [1], Diana I. Ivanova [2,*] , Yana E. Ilieva [3], Alexander N. Tashev [4],
Maya M. Zaharieva [3], George Angelov [2,*] and Hristo M. Najdenski [3]

[1] Institute of Polymers, Bulgarian Academy of Sciences, 1113 Sofia, Bulgaria; kalinova@polymer.bas.bg (R.G.K.); dimitrov@polymer.bas.bg (I.V.D.)
[2] Institute of Chemical Engineering, Bulgarian Academy of Sciences, 1113 Sofia, Bulgaria
[3] The Stephan Angeloff Institute of Microbiology, Bulgarian Academy of Sciences, 1113 Sofia, Bulgaria; illievayana@gmail.com (Y.E.I.); zaharieva26@yahoo.com (M.M.Z.); hnajdenski@abv.bg (H.M.N.)
[4] Department of Dendrology, University of Forestry, 1797 Sofia, Bulgaria; altashev@mail.ru
* Correspondence: dianadoc@abv.bg (D.I.I.); georgeangelov@yahoo.com (G.A.)

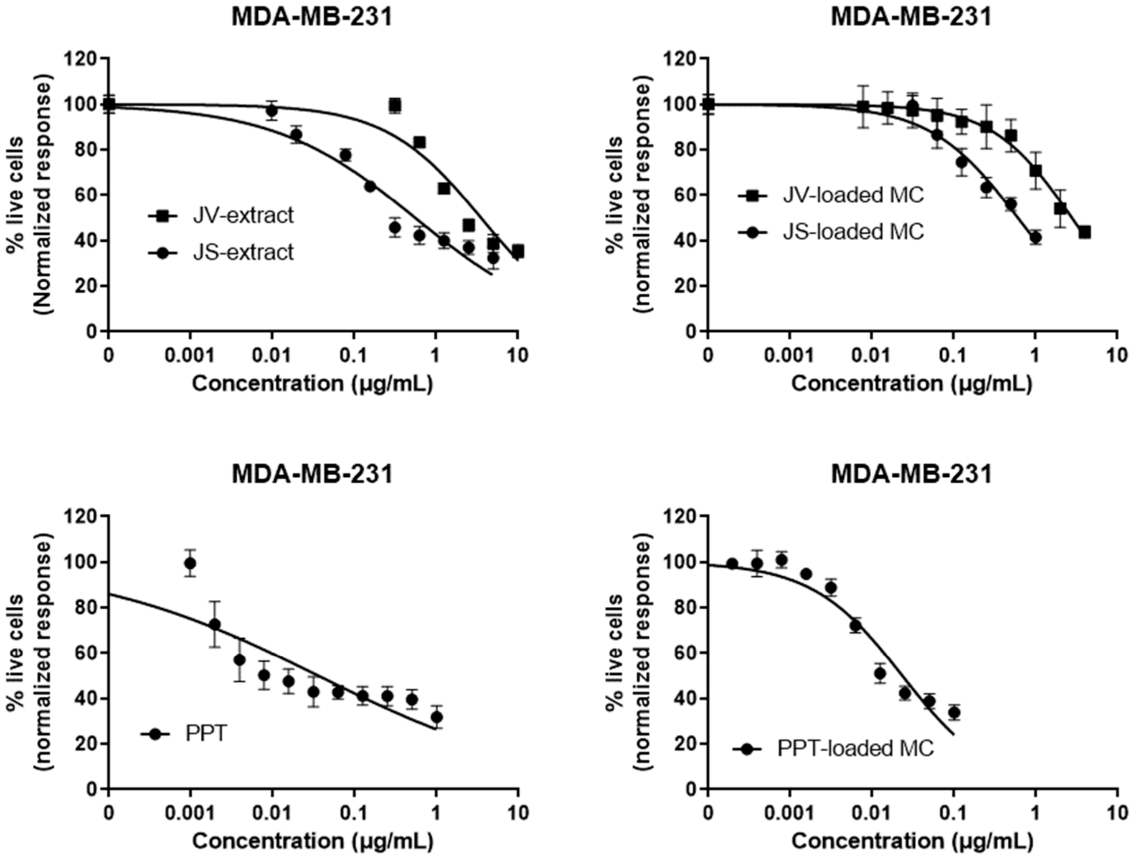

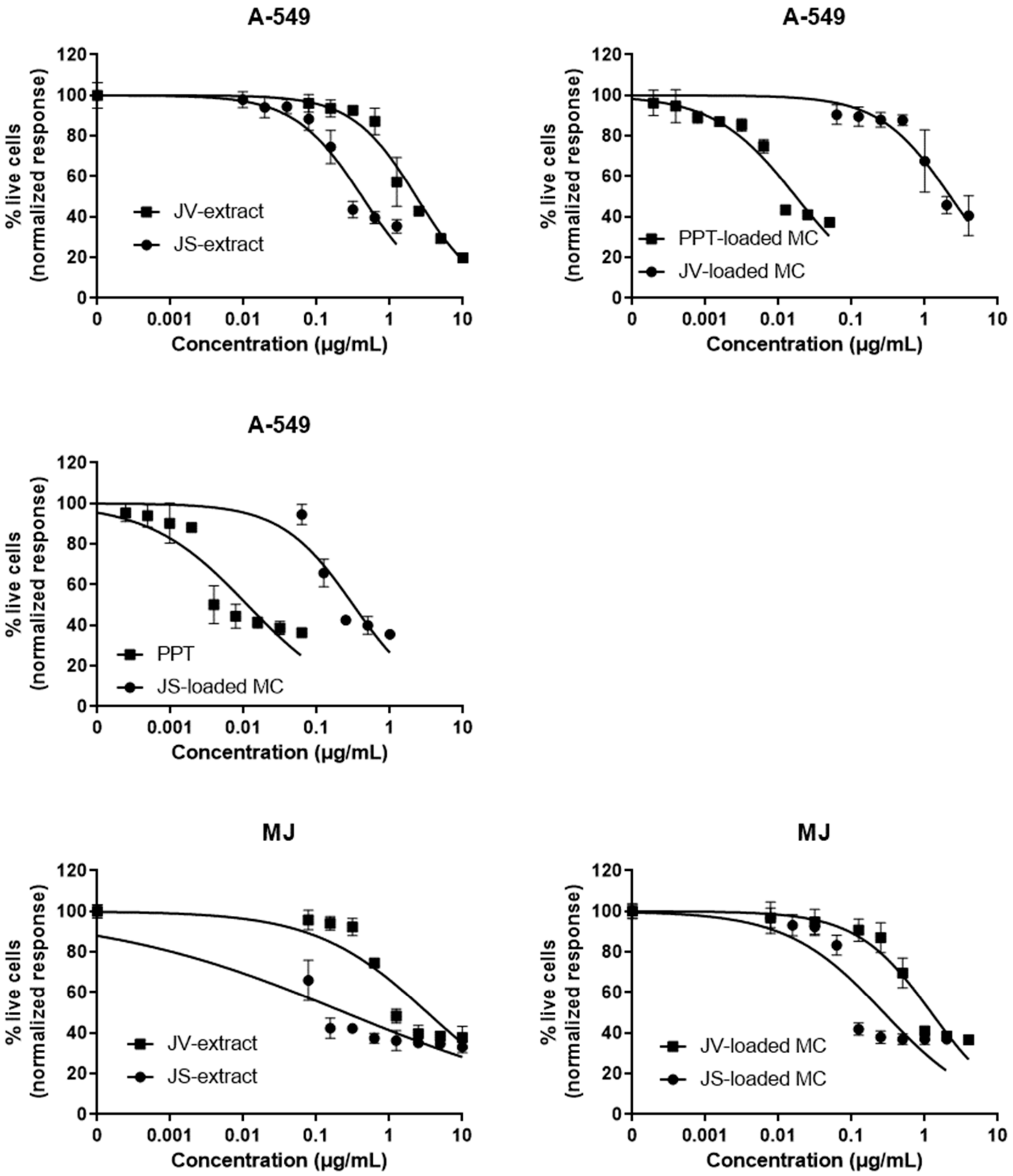

A-549
% live cells (normalized response)
Concentration (µg/mL)
JV-extract
JS-extract
A-549
PPT-loaded MC
JV-loaded MC
A-549
PPT
JS-loaded MC
MJ
JV-extract
JS-extract
MJ
JV-loaded MC
JS-loaded MC

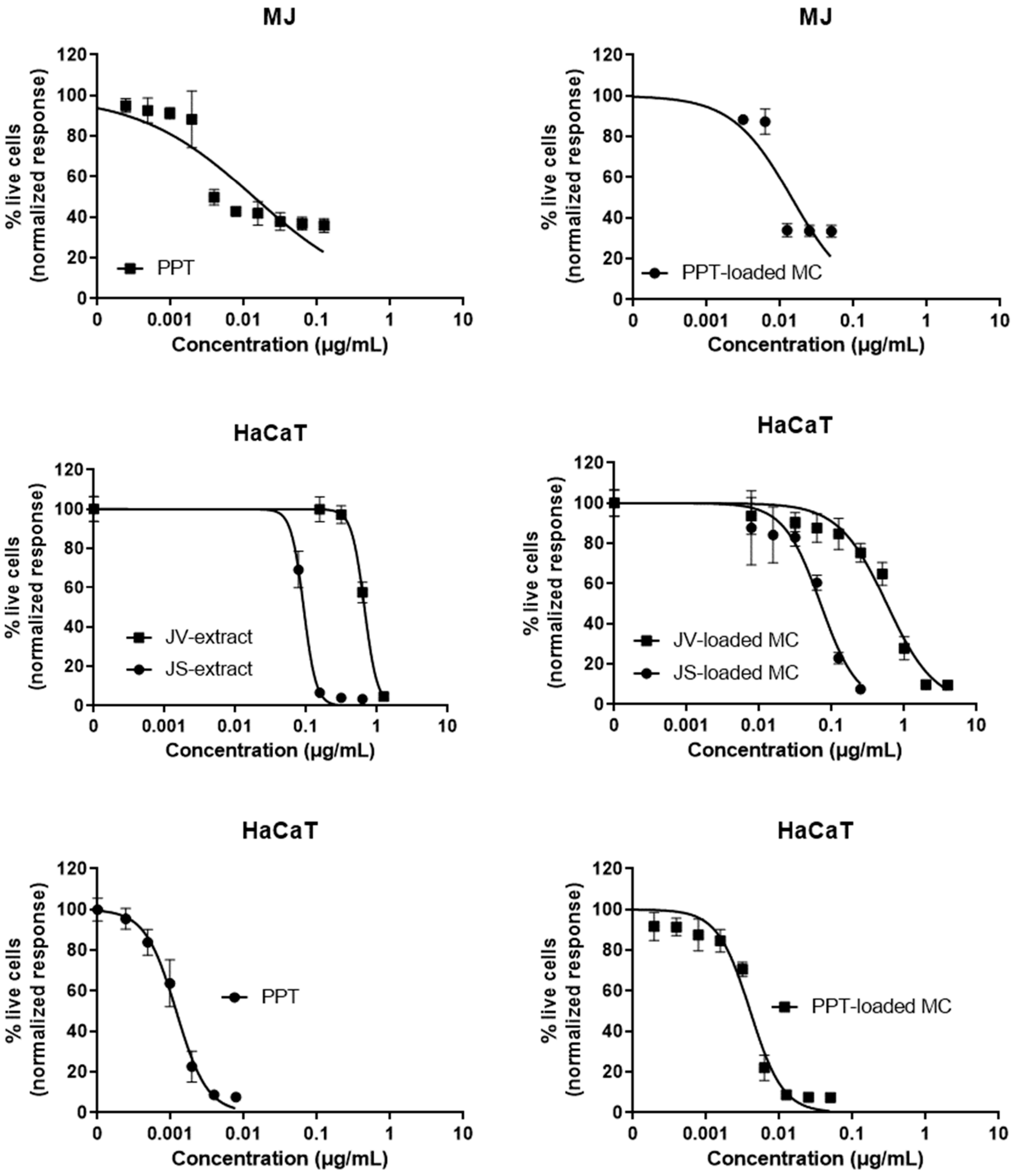

Dose-response curves derived from MTT-assays of cancer and normal cell lines treated with polymer micelles loaded with podophyllotoxin or various juniper extracts.

Abbreviations:

MC-polymer micelles;

JV - *J. virginiana* leaf extract;

JS – *J. sabina* var. *balkanensis* leaf extract;

PPT – podophyllotoxin;

MDA-MB-231 – triple negative (ER, PR, HER2 negative) breast adenocarcinoma cells;

A-549 – human lung carcinoma cells;

MJ – cutaneous T cell lymphoma cells;

HaCaT – normal human epidermal keratinocytes;
